# Midgut Mitochondrial Function as a Gatekeeper for Malaria Parasite Infection and Development in the Mosquito Host

**DOI:** 10.3389/fcimb.2020.593159

**Published:** 2020-12-11

**Authors:** Shirley Luckhart, Michael A. Riehle

**Affiliations:** ^1^Department of Entomology, Plant Pathology and Nematology, University of Idaho, Moscow, ID, United States; ^2^Department of Biological Sciences, University of Idaho, Moscow, ID, United States; ^3^Department of Entomology, University of Arizona, Tucson, AZ, United States

**Keywords:** mosquito, malaria, *Anopheles*, *Plasmodium*, mitochondria, midgut, resistance, immunity

## Abstract

Across diverse organisms, various physiologies are profoundly regulated by mitochondrial function, which is defined by mitochondrial fusion, biogenesis, oxidative phosphorylation (OXPHOS), and mitophagy. Based on our data and significant published studies from *Caenorhabditis elegans*, *Drosophila melanogaster* and mammals, we propose that midgut mitochondria control midgut health and the health of other tissues in vector mosquitoes. Specifically, we argue that trade-offs among resistance to infection, metabolism, lifespan, and reproduction in vector mosquitoes are fundamentally controlled both locally and systemically by midgut mitochondrial function.

## Introduction

Across diverse organisms, physiology is profoundly regulated by mitochondrial function, which is defined by the sum of mitochondrial fusion, biogenesis, oxidative phosphorylation (OXPHOS) and mitophagy (Zhu et al., 2013; Yu et al., 2020). Based on significant work from *Caenorhabditis elegans* (Chikka et al., 2016; Kwon et al., 2018) and *Drosophila melanogaster* (Miguel-Aliaga et al., 2018), it is likely that mitochondrial control of tissue health—and gut health in particular—is conserved across the Ecdysozoa. Importantly, the balance of mitochondrial function and dysfunction, particularly in the midgut, is highly relevant to mosquito vectors of human pathogens (Luckhart and Riehle, 2017). We hypothesize that mitochondrial activity in the midgut, the initial interface between the mosquito and *Plasmodium* parasites, dictates the balance of pathogen susceptibility and the life history traits essential for ensuring pathogen transmission. Further, the regulation of midgut mitochondrial activity is regulated in a large part through the insulin/insulin-like growth factor signaling (IIS) cascade. In this review, we will discuss data that support the inference that trade-offs among resistance to infection, lifespan and reproduction in mosquito vectors of malaria parasites are fundamentally controlled both locally and systemically by midgut mitochondrial function *via* IIS. While this mini-review will focus on mitochondrial activity in the mosquito gut, due to the central regulatory role of the gut and its unique interaction with pathogens in the bloodmeal, it is likely that mitochondrial activity in diverse tissues also has important roles in regulating the physiologies described below.

## Insulin/Insulin-Like Growth Factor Signaling Controls Mitochondrial Function to Alter Diverse Physiologies Impacting Vectorial Capacity

**Figure 1:**
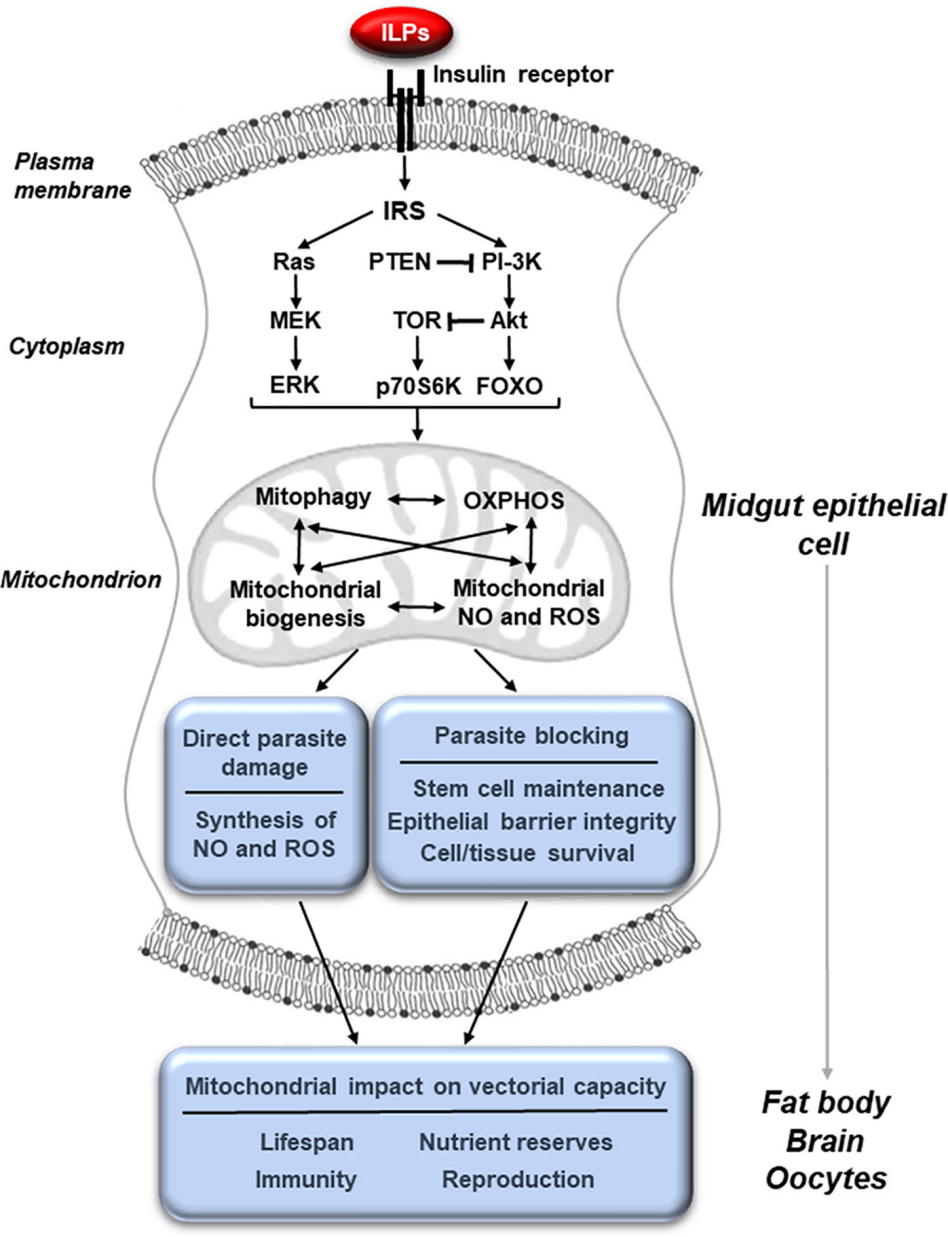
Midgut mitochondrial function, activated through IIS, controls physiologies that govern vectorial capacity. The binding of insulin-like peptides (ILPs) to the insulin receptor at the membrane of midgut epithelial cells activates the insulin/insulin-like growth factor signaling (IIS) cascade leading to changes in mitochondrial activity. These can include changes in mitophagy, biogenesis, oxidative phosphorylation (OXPHOS) and the synthesis of nitric oxide (NO) and reactive oxygen species (ROS). These mitochondrial changes can in turn directly result in parasite killing and improve the integrity of the epithelial barrier, making it difficult for the parasite to escape from the midgut cell. In addition, mitochondrial changes in the midgut epithelium can influence other tissues, altering key physiologies such as reproduction, lifespan, metabolism and immunity that affect vectorial capacity.

IIS regulates mitochondrial function across a wide range of organisms and, in this capacity, contributes to control of longevity, host immunity, cellular metabolism and the response to stress in mammals and in the model organisms *D. melanogaster* and *C. elegans*. We and others have demonstrated that IIS in adult female mosquitoes regulates egg production, longevity, defenses against infection, metabolism and the host stress response (Graf et al., 1997; Riehle and Brown, 1999; Riehle and Brown, 2002; Riehle and Brown, 2003; Lim et al., 2005; Kang et al., 2007; Roy et al., 2007; Brown et al., 2008; Sim and Denlinger, 2008; Arik et al., 2009; Corby-Harris et al., 2010; Horton et al., 2010; Gulia-Nuss et al., 2011; Marquez et al., 2011; Surachetpong et al., 2011; Pakpour et al., 2012; Hauck et al., 2013; Luckhart et al., 2013; Drexler et al., 2014; Arik et al., 2015; Pietri et al., 2016; Nuss and Brown, 2018). Further, substantial data indicate that IIS-dependent phenotypes are mediated through changes in mitochondrial function (**Figure 1**) in model invertebrates, mosquitoes and in mammals (Tóth et al., 2008; Cheng et al., 2010; Tiefenbock et al., 2010; Luckhart et al., 2013; Sadagurski and White, 2013; Drexler et al., 2014; Mukherjee et al., 2014; Pietri et al., 2016; Chaudhari and Kipreos, 2017; Ruegsegger et al., 2018; Wang et al., 2019; Wardelmann et al., 2019; Charmpilas and Tavernarakis, 2020; Sheard et al., 2020). Specifically, perturbations of both the IIS activator Akt and the inhibitor PTEN in the midgut of *Anopheles stephensi* led to profound changes in midgut mitochondrial number, quality and function as discussed below (Luckhart et al., 2013; Hauck et al., 2013).

## Mitochondrial Function is Evolutionarily Conserved and Regulated by Mitochondrial Quality Control

Mitochondrial biogenesis or the generation of new mitochondria and elimination of damaged mitochondria *via* mitophagy, a form of organelle-specific autophagy, control mitochondrial function and have profound effects on health and disease in diverse organisms (Palikaras et al., 2018). In particular, the balance between mitochondrial biogenesis and mitophagy is highly responsive to the host environment, including changes in cellular metabolism and energy status and activation of immunity to infection, to ensure tissue and organismal homeostasis (Youle and van der Bliek, 2012; Ma et al., 2020).

In general, mitochondrial damage from oxidative stress, stress-induced changes in energy status and xenobiotic insults can initiate mitochondrial fission or the separation of compromised mitochondria for removal by mitophagy (Elgass et al., 2012). The genomes of *A. stephensi* and *Anopheles gambiae* encode autophagy machinery orthologs for Parkin, PINK1, more than a dozen autophagy-related (Atg) genes and two vacuolar protein sorting genes. In brief, damaged mitochondria are targeted for the aggregation of PINK1, which recruits the E3 ubiquitin ligase Parkin to activate Atg proteins to form the autophagosome. Activators of autophagy include mitochondrial reactive oxygen species (ROS) as well as signaling through ERK-dependent and p38 MAPK and AMP-activated protein kinase (AMPK; Huang et al., 2011; Levine et al., 2011; Corona Velazquez and Jackson, 2018). Autophagy is inhibited by signaling through the PI-3K/Akt branch of the IIS cascade, signaling that also controls mitochondrial biogenesis (Huang et al., 2011; Levine et al., 2011; Denton et al., 2012; Manning and Toker, 2017). Specifically, when FOXO is excluded from the nucleus by PI-3K/Akt signaling, PPAR gamma coactivator-1 alpha (PGC-1), a key mediator of mitochondrial biogenesis, is not activated (Cheng et al., 2009). Conversely, overexpression of the IIS inhibitor PTEN, which results in FOXO translocation into the nucleus, induces PGC-1 activity (Garcia-Cao et al., 2012). In *D. melanogaster*, FOXO-induced PGC-1 increased levels and activity of electron transport chain (ETC) complexes I, II, and IV, indicating that enhanced functioning of the ETC is positively associated with biogenesis (Rera et al., 2011). Increased midgut mitochondrial biogenesis in *D. melanogaster* resulted in improved midgut epithelial integrity, and we have associated this biological dynamic in *A. stephensi* with reduced development of the human malaria parasite *Plasmodium falciparum* in the absence of enhanced NF-κB-dependent immune gene expression (Rera et al., 2011; Pakpour et al., 2012; Hauck et al., 2013; Pakpour et al., 2013). Mitochondrial biogenesis, which is activated by NO-dependent guanylate cyclase as well as by AMPK signaling, was hyperactivated and out of balance with mitophagy in *A. stephensi* engineered to overexpress activated Akt in the midgut epithelium (Nisoli et al., 2003; Nisoli et al., 2004; Nisoli et al., 2005; Fernandez-Marcos and Auwerx, 2011; Luckhart et al., 2013). While Akt-dependent mitochondrial dysfunction was deleterious to *A. stephensi*, it also conferred extreme resistance to *P. falciparum* infection (Corby-Harris et al., 2010; Luckhart et al., 2013).

## Mitochondrial Function Controls Epithelial Barrier Integrity and Stem Cell Activity in The Gut

An analysis of mitochondrially controlled phenotypes is informative for the idea that mitochondria are master regulators of key physiologies in mosquitoes (**Figure 1**). When AMPK was overexpressed in the midgut of *D. melanogaster*, the age-related decline in epithelial barrier integrity was reduced, indicating that both autophagy and mitochondrial biogenesis protected the integrity of the midgut barrier (Rera et al., 2011; Ulgherait et al., 2014). In more recent work, treatment with rapamycin, which inhibits Akt-dependent mechanistic Target of Rapamycin (mTOR) signaling, maintained epithelial barrier integrity and extended lifespan in *D. melanogaster* independently of any changes in the gut microbiota (Schinaman et al., 2019). In mammals, AMPK is required for regulation of polarity of hepatocytes and other epithelial cell types as well as tight junction and barrier integrity through increased mitochondrial fusion and biogenesis (Treyer and Müsch, 2013; Kang et al., 2016; Ghosh, 2017). Successful *P. falciparum* infection of hepatocytes has been associated with reduced AMPK activity in these cells, whereas pharmacological induction of hepatocyte AMPK reduced intracellular parasite growth (Ruivo et al., 2016). In our work, we observed increased midgut barrier integrity in transgenic (TG) *A. stephensi* with increased PTEN expression in the midgut, which limited *P. falciparum* infection without changes to NF-κB-dependent anti-parasite gene expression (Hauck et al., 2013). Since the IIS activator Akt can suppress both mitochondrial biogenesis and autophagy, it is likely that overexpression of the IIS inhibitor PTEN in *A. stephensi* increased autophagy, mitochondrial fusion and mitochondrial biogenesis to enhance midgut barrier integrity, in turn increasing mosquito resistance to *P. falciparum* infection (Huang et al., 2011; Levine et al., 2011; Denton et al., 2012; Hauck et al., 2013). Supporting this idea, biomarker expression for both midgut stem cells (MSC) and autophagy were increased in TG *A. stephensi* overexpressing PTEN relative to non-transgenic (NTG) controls (Hauck et al., 2013). Collectively, our data and these observations suggest that manipulation of pathways leading to increased midgut mitochondrial fusion and biogenesis have the potential to improve midgut epithelial integrity and in turn resistance to *P. falciparum* infection in *A. stephensi*.

A proper balance between mitophagy and mitochondrial biogenesis is essential for optimal stem cell differentiation and maintenance. Previous studies have demonstrated the important roles stem cells play in the midgut of insects, including mosquitoes (Brown et al., 1985; Baton and Ranford-Cartwright, 2007). Intriguingly, Janeh et al. (2019) reported that, in contrast to *Aedes albopictus* and *Culex pipiens*, midgut tissue in *A. gambiae* does not contain cells that undergo mitosis to repair gut damage. The authors speculated that this difference could underlie permissiveness to malaria parasite infection and rightly suggested that additional mechanistic studies are necessary to fully understand the biological importance of these differences across mosquito genera. In *D. melanogaster*, upregulation of mitochondrial biogenesis in the midgut prevented MSC over-proliferation and the accumulation of improperly differentiated cells (Rera et al., 2012a). Coupled with an enhanced epithelial barrier, this phenotype confirmed that reduced MSC dysplasia could extend lifespan. When *A. stephensi* were provisioned with human IGF1 in the bloodmeal at concentrations consistent with titers found during malaria, midgut cytoprotection was enhanced through the homeostatic regulation of ROS and patterns of gene expression consistent with both MSC activity and autophagy, resulting in improved mitochondrial function and a more robust midgut epithelium (Drexler et al., 2014). These data suggested that enhancing mitochondrial function to increase midgut health could improve both parasite resistance and lifespan in *A. stephensi* (Drexler et al., 2014). In addition to these processes, the Hippo pathway regulates mitochondrial function in both human and *D. melanogaster* cells (Nagaraj et al., 2012). In *D. melanogaster*, knockdown of Hippo or overexpression of Yorkie, the transcriptional effector of the pathway, enhanced MSC proliferation and prevented apoptosis, providing additional support for the role of mitochondrial function in epithelial homeostasis (Huang et al., 2005; Ren et al., 2010).

## Mitochondrial Function Controls Diverse Physiologies That are Key to Vectorial Capacity

Host immunity to infection is regulated by mitochondrial function. Increased biogenesis of mitochondria during infection, possibly in response to cell injury, can alter the production of pro- and anti-inflammatory cytokines to improve mammalian host convalescence and survival (Sweeney et al., 2010; Carré et al., 2010; Sweeney et al., 2011; Tran et al., 2011; Dada and Sznajder, 2011; Piantadosi et al., 2011). ETC Complex I- and III-generated ROS can also activate Toll-like receptor signaling and NF-kB-dependent gene expression for the targeted elimination of pathogens (Arnoult et al., 2011). In *A. stephensi*, activation of IIS induced high levels of mitochondrial anti-parasite ROS and NO, responses that also resulted in significant damage to the midgut epithelium (Luckhart et al., 2013). This damage, however, can be mitigated by parasite-induced p38 MAPK signaling and associated increases in mitochondrial biogenesis, oxidative phosphorylation (OXPHOS) and antioxidant biosynthesis for mosquito survival during infection (Wang et al., 2015). In an effort to establish cause-and-effect of these metabolic changes with infection outcome, we provisioned small molecule inhibitors to *A. stephensi* to demonstrate that midgut intermediary metabolism, in fact, regulates *P. falciparum* infection (Pietri et al., 2016). Notably, these small molecule inhibitors had no effect on NF-kB-dependent immune gene expression, thereby linking anti-parasite resistance to shifts in glycolysis and mitochondrial function (Pietri et al., 2016). To our knowledge, this was the first evidence in anopheline mosquitoes that direct manipulation of metabolism and mitochondrial function could alter anti-parasite immunity.

Longevity is one of the most complex phenotypes regulated by mitochondrial physiology and is a life history trait intimately connected to resistance to infection across a wide range of organisms. Specifically, modest reductions in mitochondrial ETC activity and OXPHOS can enhance survival in organisms ranging from yeast to mice (reviewed in Rera et al., 2012a). A modest *increase* in ETC activity, however, can be effective as well. When ETC Complex I activity was increased in *D. melanogaster* through the overexpression of yeast NADH-ubiquinone oxidoreductase the integrity of the midgut barrier was improved and lifespan extended, without a reduction in fecundity (Hur et al., 2013). The fact that both activation and inhibition of ETC Complex I can lead to increased longevity is an example of hormesis, where modest positive and negative changes can both drive an organism to improved mitochondrial function (Hur et al., 2014). Increased autophagy has also been found to extend lifespan of *D. melanogaster*. In this context, overexpression of Parkin, a key mediator of mitophagy, significantly extended lifespan *and* increased fruit fly fecundity (Rana et al., 2013). In contrast, mutations in PINK1, which binds to damaged mitochondria and recruits Parkin, were associated with a reduction in lifespan (Clark et al., 2006). In our work, PTEN overexpression in the *A. stephensi* midgut increased longevity, enhanced the midgut barrier, improved *P. falciparum* resistance and had no negative impact on fecundity (Hauck et al., 2013). This work suggested that PTEN overexpression in *A. stephensi* and the anticipated downstream effects on both PINK1 and Parkin improved mitochondrial quality control, which led to increased longevity and parasite resistance (Unoki and Nakamura, 2001). Extensive studies on AMPK in diverse organisms, including *D. melanogaster* and *A. aegypti*, demonstrated a key role for this protein in the regulation of senescence as well. In *D. melanogaster*, upregulation of AMPK in the midgut significantly extended lifespan (Ulgherait et al., 2014). In *A. aegypti*, a diet supplemented with polyphenols increased lifespan, in part through AMPK activation (Nunes et al., 2016). Our studies, however, did not replicate polyphenol-dependent life extension in *A. stephensi*, suggesting that these two mosquito species may have intriguing differences in longevity regulation (Johnson and Riehle, 2015).

Reproduction in mosquitoes, unlike most other Diptera including *D. melanogaster*, is intermittent and dependent on nutrient-rich bloodmeals, which initiate a reproductive cycle. This reproductive cycle, including oogenesis and vitellogenesis, is tightly regulated through a combination of hormonal signals, signaling cascades and nutrient sensors. In response to the blood-filled midgut, signals from the brain including insulin-like peptides (ILPs) and ovarian ecdysteroidogenic hormone (OEH) induce ovarian synthesis of 20-hydroxyecdysone (20E), which triggers fat body biosynthesis of lipids and other nutrients for egg development. The impact of 20E on malaria parasite infection is complex, with two studies reporting that 20E increases resistance of *A. gambiae* to *P. falciparum* and *Plasmodium berghei* (Upton et al., 2015; Reynolds et al., 2020). In contrast, Dahalan et al. (2019) showed that male-derived 20E, delivered to the female mosquito during mating, dramatically altered the midgut transcriptome in patterns distinct from those of systemic effects of 20E and decreased resistance of *Anopheles coluzzii* to *P. falciparum*. In addition to IIS regulation of 20E, IIS regulation of mitochondrial dynamics governs reproductive success in *D. melanogaster* while mitochondrial variation-dependent outcomes in innate immunity of fruit flies likely also drives immunity-fecundity tradeoffs. Notably, ubiquitous and neuron-specific overexpression of Parkin in adult fruit flies led to a significant increase in fecundity, in addition to lifespan extension (Rana et al., 2013). In a recent review, Salminen and Vale (2020) elegantly argue that variation in mitochondrial function of *D. melanogaster*, which has many hallmarks of the same biology in mammals, likely drives variation in fruit fly innate immune function. By extrapolation, this variation, which ranges from optimal mitochondrial dynamics to extreme dysfunction, would be predicted to underlie observed tradeoffs between immunity and fecundity observed in fruit flies with abnormal mitochondrial energy metabolism (Buchanan et al., 2018). Our own work in *A. stephensi* demonstrated that Akt signaling directly influences mitochondrial activity in the midgut (Luckhart et al., 2013). Further, increased Akt signaling in the fat body of *A. stephensi*, which most likely influences mitochondrial activity in this tissue as well, led to a significant increase in lifetime fecundity (Hun et al., 2019). In light of these observations, it is likely that IIS/TOR-dependent mitochondrial activity controls reproduction in *Anopheles* spp., but we would add that underlying IIS-dependent mitochondrial variation likely also drives the somewhat puzzling range of tradeoffs that are evident during infection.

## Conclusion

The *C. elegans* and *D. melanogaster* guts are among the most stress sensitive tissues and function as critical “signaling centers” for communicating changes in mitochondrial function (Alic et al., 2014; Owusu-Ansah and Perrimon, 2015; Liu and Jin, 2017). For example, aging in *D. melanogaster* has been associated with a general decline in tissue mitochondrial biogenesis, but manipulations to *systemically* increase mitochondrial biogenesis had no effect on fruit fly longevity. However, targeted enhancements in *gut-specific* mitochondrial biogenesis enhanced *D. melanogaster* longevity *via* coordinated changes in tissue mitochondrial function (Vellai, 2009; Rera et al., 2012b; Ulgherait et al., 2014). Notably, gut mitochondrial biogenesis was associated with an improved midgut epithelial barrier, preventing lumenal microbes from initiating disseminated infections (Rera et al., 2012b). Accordingly, manipulation of *gut* mitochondrial function in *D. melanogaster* can enhance immunity locally and fitness systemically. Importantly, mitochondrial changes in fat body and muscle can contribute independently to these phenotypes as well, indicating that local mitochondrial changes induce *functional* systemic changes to extend local effects (Owusu-Ansah and Perrimon, 2015). Based on these observations, our data and the work of others, we hypothesize that changes in midgut mitochondrial dynamics function both *locally* and *systemically* to coordinate pathogen resistance, metabolism, lifespan and reproduction in *Anopheles* mosquitoes (**Figure 1**).

Is the *Anopheles* midgut a signaling center for mitochondrial physiology and the coordination of changes in host biology during infection? Published data and our data argue that this is case, but considerable work remains to be done in understanding this biology and its influence on mosquito resistance to infection, networking of life history traits critical to vectorial capacity and the dependency of this network on the coordination of mitochondrial function among the midgut and other tissues. Will this biology simply be a carbon copy of what is known from model invertebrates and mammals? If viewed *only* from the perspective of gene conservation, the answer is likely to be yes. However, the relevance of this biology to mosquitoes is uniquely elevated, defined and dictated by bloodfeeding and the consequences to human health that are vastly different from other organisms. Accordingly, this biology has both translational importance to “getting over the gut” and critical relevance to our basic scientific knowledge, with likely twists and turns that will provide a fascinating and substantive extension of our general knowledge of mitochondrial control of animal physiology.

## Author Contributions

SL and MR wrote and revised this mini-review. All authors contributed to the article and approved the submitted version.

## Funding

The authors’ work cited here has been supported by the National Institutes of Health, National Institute of Allergy and Infectious Diseases awards R01AI060664, R01AI080799, R56AI107263, R56AI118926, R56AI129420, R01AI073745, and R21AI125823.

## Conflict of Interest

The authors declare that the research was conducted in the absence of any commercial or financial relationships that could be construed as a potential conflict of interest.

## Abbreviations

20E, 20-hydroxyecdysone; Akt, IIS kinase, also known as protein kinase B; AMPK, 5’ adenosine monophosphate-activated protein kinase; Atg, autophagy; ERK, extracellular signal-regulated kinase; ETC, electron transport chain; FOXO, class O forkhead box transcription factors; IGF1, insulin-like growth factor 1; IIS, insulin/insulin-like growth factor signaling; ILP, insulin-like peptide; IRS, insulin receptor substrate; MEK, mitogen-activated protein kinase kinase; MSC, midgut stem cells; mTOR, mechanistic target of rapamycin; NADH, reduced form of nicotinamide adenine dinucleotide; NF-κB, nuclear factor-kappa B; NO, nitric oxide; NTG, non-transgenic; OEH, ovarian ecdysteroidogenic hormone; OXPHOS, oxidative phosphorylation; p38 MAPK, p38 mitogen-activated protein kinase; p70S6K, p70 S6 kinase; PGC-1, peroxisome proliferator-activated receptor gamma coactivator-1; PI-3K, phosphatidylinositol-3-kinase; PINK1, PTEN-induced kinase 1; PTEN, IIS protein, also known as phosphatase and tensin homolog; ROS, reactive oxygen species; TG, transgenic.
